# The Newness of Dentistry

**Published:** 1900-06

**Authors:** Charles W. Eliot


### THE NEWNESS OF DENTISTRY.[1]

BY CHARLES W. ELIOT, LL.D.[2]

MR. PRESIDENT AND GENTLEMEN,—The coming together of these two societies is interesting to me, because I find the same sort of thing going on all over the country, in all branches of education, and in all the professions, scientific and learned. It is a new phenomenon, this rapid formation of sympathetic and co-operative societies. I have seen it within ten days in a new departure among the American universities. In Chicago, delegates from fourteen universities decided that they would begin the establishment of an Association of American Universities. We have never had such a thing before. We do not know exactly what will come out of it; but we are sure, those of us who took part in this beginning, that we shall gain through the co-operation of American universities an influence which the separate institutions would never have been able to exert. Just so, in the various recent societies which have been formed in this country in each of the learned and scientific professions, the co-operation of men of the same calling in different parts of the country, through societies which have common objects though perhaps different local problems, is, I believe, a new sign of progress.

The President has indicated the subject to which my name is attached in the programme, "The Newness of Dentistry." It is, I believe, the newest of the callings which require a high degree of knowledge and technical skill. It has taken advantage in the most rapid way of many of the most recent discoveries in chemistry, physiology, physics, and mechanical engineering. It has taken advantage of many of the interesting novelties in metallurgy and in the application of different combinations of metals. It has profited by the innumerable inventions and discoveries into which India rubber and gutta-percha enter. The laboratory of a dental practitioner to-day seems to me to be an epitome of applied chemistry, physiology, bacteriology, metallurgy, engineering, and physics, to which we must add the latest improvements in surgery. I need

---

[1] Read before the union meeting of The New York Institute of Stomatology and the American Academy of Dental Science, Boston, March 21, 1900.

[2] President of Harvard University.

but to enumerate these topics to show, in the first place, what a wide range of knowledge a dentist needs; secondly, what various skill he needs; and, thirdly, how new almost all his means and methods must be, because these sciences on which he relies are new to the world. A dentist, then, is a pioneer in the application of science; and he is a pioneer in a large region, because he relies upon the exact physical sciences quite as much as upon the sciences which are grouped under the general name of Natural History. He has a great field.

I remember, when the Dental School was first organized in Harvard University, that some of the advisers of the Corporation said, "Dentistry is too small a subject for a real profession,—it only deals with the mouth. It has too small a field for a university school." Fortunately, this somewhat narrow doctrine did not prevail, but it was warmly maintained in the discussion which preceded the foundation of our Dental School. It never seemed to me to be well founded. When we imagine treating with success any part of the human body, do we not invariably find it difficult to define the limits of the knowledge and skill which may be useful in that treatment? The specialists of the body, the specialists of any part of the body, need to be thoroughly informed men. If they are not, they cannot have the highest success in their specialties. It must be so in dentistry; yet it must be confessed that this is a comparatively new view of dentistry. Here again we have one of the aspects in which dentistry is a new profession.

In one respect our country has failed in its treatment of the dental profession. The dental schools taken together have not insisted upon a good education before the professional training was entered upon. This defect of dental education in our country is, I believe, due to a too limited view of the function of the dentist after he has entered upon his profession. I should be glad to think that these two societies would contribute to remedy this evil. I have been much impressed, during the striking development of the medical profession within the last thirty years in this country, with the great importance of the combined sentiment of medical men towards the reform and improvement of their profession; and I believe that a similar influence can be relied on for the improvement of the profession of dentistry. We can rely upon the dentists' interest in their calling to elevate the standard of the preliminary education for the profession.

The confidence of the community in the profession, and particularly the interest of the medical profession in the calling of the dentist, would, I think, be promoted if the dental profession as a whole entered more than it does into the problems of medical investigation. The dentist seems to me to have some remarkable facilities for contributing to the progress of preventive medicine. I remember that forty-five years ago or thereabouts, being in Richmond, Va., one day, I went into the slave market, curious, as a Northerner might naturally be, to see how men, women, and children were bought and sold; and I watched the auction for an hour or more. The first thing that struck me was the reliance placed by the purchasers upon the examination of the teeth. Everybody who is buying a horse or a dog looks at the teeth, partly for the determination of age, but also to determine the probable constitution and vigor. That is just what the buyers did to human beings in the Richmond slave market. Now, a dentist who practises for forty years sees, or may see, three generations of the same family; and if it were the habit of the profession to record the facts about the teeth of these generations, and the evidence the teeth give concerning diet and health habits, should we not learn something valuable about diet and the effects of different diets upon the constitution of the human creature? Surely we desperately need information on that subject.

My attention was drawn to this subject many years later, when I became pretty well acquainted with the population of the coast of Maine. I used to go yachting along that coast every summer; and the pleasure of yachting is being in with the land. The ocean is all very well; but one wants to go into harbor every night to have a quiet anchorage and see a new place. Therefore, I visited the villages all along the coast and up the rivers; and I was very much struck with the bad condition of the teeth of the young people. The generation preceding had very good teeth. If the slave market had prevailed along the coast of Maine in the last twenty-five years, the purchasers would have had very great difficulty in determining the value of the young men and women put up for sale; for their teeth were gone. What was the cause of this ruin? I believe it rested entirely upon the change in the diet of the population which resulted from the importation into Maine of fine white flour, —the finest possible white wheat flour was what the native bought, and he added to that sugar and what he called sauce, and, with doughnuts and sweet cakes, these were his staple foods. Now, it did not take more than twenty or twenty-five years of this diet to change the teeth of the population very much for the worse. The previous generation used the wheat raised on their own farms and ground at the neighboring mill; and so they ate the whole wheat. I mention this simply to illustrate what I believe would be the very valuable contribution which dentists might make to our knowledge of the safe diet for a population become largely urban. I think we ought to hope to receive from the dental profession as a whole important contributions to medical research, and to our knowledge of the human body in health, and of the conditions under which health may be preserved.

The dental profession uses many new materials, new methods, and new powers, and I think may justly be described as new within a hundred years, perhaps within fifty years; but sometimes I think everything has been made new within the century now drawing to its close. A Chicago man was telling me the other day, when I mentioned certain slightly barbaric features of the city, that a few leading citizens had had to create everything in Chicago within the last fifty years. Fifty years ago it was nothing but a mud-hole, and now it was a great city; and a comparatively small number of men had created all the institutions of the place. I asked him if he supposed that Boston differed from Chicago in that respect. He said he did; but I replied, "There is not a thing in Boston that has not been made over root and branch within forty years; our water supply, our sewerage system, all our means of heating and lighting, and all our methods of transportation,—everything has been made over in the city of Boston." If you look abroad over the country, and see the condition of our government, of our schools and colleges, of our public institutions, our hospitals, libraries, and churches, they are either new or have been made over. The extreme novelty of all the grave problems that press upon our communities is, I believe, the fundamental reason why we have such great difficulty in dealing with them.

One of the great changes in the last twenty years is the rush of our populations into cities and towns. That is a change that has affected strongly the practice of dentistry. Dentistry is an art for an urban population, and our population becomes more and more urban. It is an art that is exercised chiefly for the benefit of the well-to-do, though we are all happy to see that the long-established usage of the medical profession, the giving of a large portion of the time of medical men to gratuitous practice, is taking a strong hold among dentists.

If, then, the dental profession is new, it shares this quality with almost all other human interests. I look forward with confidence to rapid and important progress in the profession which you represent here so honorably. Indeed, I have personally seen such rapid advance, so many new fields conquered within the last thirty years, that I should be faithless, indeed, if I did not anticipate with confidence a vigorous development in the future.

